# Case Report: Lenvatinib for the treatment of recurrent hepatocellular carcinoma in people living with HIV: a report of two cases

**DOI:** 10.3389/fonc.2023.1242741

**Published:** 2023-12-05

**Authors:** Giulia Morsica, Costanza Bertoni, Hamid Hasson, Emanuela Messina, Caterina Uberti Foppa

**Affiliations:** ^1^ Department of Infectious Diseases, IRCCS, San Raffaele, Scientific Institute, Milan, Italy; ^2^ Faculty of Medicine and Surgery, Vita-Salute University, Milan, Italy

**Keywords:** hepatocellular carcinoma, people living with HIV, lenvatinib, recurrent, therapy

## Abstract

The use and choice of the best systemic treatment is gaining increasing interest in people living with HIV (PLWH) because hepatocellular carcinoma (HCC) represents an increasing cause of morbidity and mortality in this setting and most HCCs are diagnosed in the advanced stage. Ten years ago, the multi-kinase inhibitor lenvatinib was approved in the first-line setting. However, to date, no data on the efficacy and tolerability of lenvatinib in PLWH from clinical trials and real-life studies are available. Case 1 was a gentleman with hepatitis B virus–related cirrhosis who underwent orthotopic liver transplantation for HCC and developed peritoneal metastasis several years later. Lenvatinib treatment was selected at HCC recurrence. This participant maintained undetectable HIV viremia and a relatively preserved immune status during 6 months of systemic treatment with lenvatinib. After 6 months, he discontinued lenvatinib for progression of the disease (growing of peritoneal metastasis) and uncontrolled hypertension. Case 2 was a gentleman with hepatitis C–genotype 1a–related cirrhosis who experienced unresectable recurrences after radiofrequency thermal ablation of the tumor. At the first recurrence, HCC was treated with six cycles of trans-catheter arterial chemoembolization; at the second recurrence, the participant underwent trans-catheter arterial radioembolization; and at the third recurrence, he received lenvatinib. A week after the start of lenvatinib, the participant had liver decompensation and discontinued therapy. The presently reported cases showed low tolerability of systemic therapy with lenvatinib in PLWH. Cumulative data are necessary to define the position of lenvatinib in this setting.

## Highlights

• This case report describes the first reported use of lenvatinib in two participants with unresectable HCC recurrence.
• Although the presently reported two cases had a good liver functional reserve and an intermediate BCLC score, they discontinued lenvatinib because of low tolerability.
• The presently reported cases underline the need to provide cumulative data on PLWH with unresectable HCC, addressing the choice between sorafenib and lenvatinib in this setting.

## Introduction

The tyrosine kinase inhibitor sorafenib was the first drug approved for advanced hepatocellular carcinoma (HCC). After 10 years, the multi-kinase inhibitor lenvatinib was approved in the first-line setting. The phase 3 REFLECT trial established the non-inferiority of lenvatinib compared with sorafenib in terms of overall survival, whereas exploratory analysis suggested a potential benefit of lenvatinib over sorafenib for individuals with HBV chronic infection and a positive alpha-fetoprotein (AFP) value (1). After the result of the phase 3 trial, several real-life studies aimed to integrate the study from the phase 3 trial, highlighting a possible superiority of lenvatinib in participants in an early and intermediate stage compared to sorafenib (2–5). The use and the choice of the best systemic treatment are of increasing interest in the setting of people living with HIV (PLWH), because HCC represents an increasing cause of morbidity and mortality in this group of participants. Because, in PLWH, most HCCs are diagnosed in the advanced stage, the systemic therapy is the only e-treatment available.

Data were collected as part of routine clinical care and recorded in the database of the Division of Infectious Diseases of the San Raffaele Hospital (CSLHIV Cohort). The CSLHIV Cohort was approved by the ethics committee of the San Raffaele Hospital, Milan, Italy. On their first visit, participants provide a written informed consent on the use of their data in scientific analyses. Recorded data are anonymized and managed according to the Good Clinical Practice.

## Case presentation

### Case 1

The participant was a 59-year-old gentleman with hepatitis B virus–related cirrhosis who was diagnosed with HCC in April 2013. In February 2014, he had undergone orthotopic liver transplantation (OLT) for HCC beyond the Milan criteria. The clinical characteristics of Case 1 before OLT and before starting HCC systemic treatment are summarized in **Table 1**. At OLT, he had a relatively preserved immune status assessed by blood chemistry (CD4 cell count of 439 cells/mmc and CD4/CD8 ratio of 1.09), and HIV viremia was undetectable (<50 copies/mL). Transaminases [aspartate aminotransferase (AST) and alanine aminotransferase (ALT) values] were within the normal range; bilirubin was slightly increased, and AFP levels were higher (27.5 ng/mL) than the normal values. After a period of relatively good clinical condition, in June 2020, the participant experienced a recurrence of HCC due to peritoneal metastasis. Therefore, systemic therapy with lenvatinib was selected in July 2020. The participant received 12 mg of lenvatinib according to body weight (≥60 kg) given once daily, orally. Before systemic therapy, his liver function was preserved: Child–Pugh–Turcotte (CPT) score was A, and the Barcelona Clinic Liver Cancer (BCLC) stage was B. He was not suffering from hypertension and had a good immune status assessed by CD4 cell count (number of cells of 908/mmc) and CD4/CD8 ratio (1.19); HIV-RNA replication (HIV-RNA < 50 copies/mL) and HBV-DNA levels (<10 IU/mL) were well controlled by antiretroviral treatment (ART) including an integrase strand transfer inhibitor (INSTI) (i.e., raltegravir) and nucleos(t)ide analogs (i.e., emtricitabine plus tenofovir alafenamide) (these latter two drugs are active on both HBV and HIV), whereas AFP was found higher than the normal values (20.2 ng/mL). During lenvatinib therapy, the participant maintained undetectable HIV-RNA and HBV-DNA levels as well as a good immune status (CD4 cell number of 785/mmc and CD4/CD8 ratio of 1.17) without liver decompensation. He discontinued lenvatinib after 6 months of treatment as a result of repeated episodes of hypertension (grade 3 hypertension) refractory to optimal antihypertensive and concomitant progression of peritoneal metastasis (during this period, treatment dosages were not reduced or interrupted). At this time point, the treatment choice was the best supportive care.

**Table 1:** Characteristics of two participants before treatment with lenvatinib.

Case 1	Before OLT	Before lenvatinib	Case 2	Before RFTA	Before TACE (first three cycles)	Before TARE	Before lenvatinib
Age, years	59	–		58	–	–	–
Years of HIV infection	22	–		31	–	–	–
CD4 number cells/mmc	439	908		623	437	445	282
CD8 number cells/mmc	402	760		ND	445	ND	276
CD4/CD8 ratio	1.09	1.19		ND	0.98	ND	1.02
Nadir CD4 number cells/mmc	228	–		310	–	–	–
HIV-RNA, copies/mL (limit of detection 50 copies/mL)	<50	<50		<50	<50	<50	<50
ART treatment	RAL FTC/TDF	RAL FTC/TAF		DAR/RTV ABC/3TC	DAR/RTV ABC/3TC	DTG FTC/TAF	DTG FTC/TAF
AST levels, IU/L	52	26		105	39	43	48
ALT levels, IU/L	29	25		134	37	36	43
Bilirubin, mg/dL	1.21	0.35		0.78	0.58	0.74	0.83
Neutrophil number cells, 10^9^/L	3.1	3.0		4.2	4.0	3.7	3.1
Lymphocyte number cells, 10^9^/L	1.3	2.5		1.9	1.3	1.2	1.0
Creatinine, mg/dL	0.94	1.34		0.82	1.05	0.97	1.3
Albumin, gr/L	34.39	43.62		41.59	38.18	39.2	40
Platelets count × 10^9^/L	63	123		128	101	87	65
AFP, ng/mL	27.5	20.2		20.1	75.6	242	5.4
Child–Pugh–Turcotte score	A	A		A	A	A	A
BCLC classification	0	B		0	B	B	B
PTINR	1.20	1.13		1.02	1.19	1.23	1.25

RFTA, radiofrequency thermal ablation; TACE, trans-catheter arterial chemoembolization; TARE, trans-catheter arterial radioembolization; RAL, raltegravir; FTC, emtricitabine; TAF, tenofovir alafenamide; DAR, darunavir; RTV, ritonavir; ABC, abacavir; 3TC, lamivudine; DTG, dolutegravir; AST, aspartate amino transferase (normal value < 35 IU/L); ALT, alanine amino transferase (normal value < 59 IU/L); AFP, alpha-fetoprotein (normal value < 4.5 ng/mL); BCLC, Barcelona Clinic Liver Cancer; PTINR, prothrombin, international normalized ratio.

In April 2022, the participant was still alive with a relatively preserved immune status, a good control of HIV viremia, and a slow progression of peritoneal metastasis.

In January 2023, the participant deceased for the progression of the disease.

### Case 2

The participant was a 58-year-old gentleman with HCV-genotype 1a–related cirrhosis and diagnosed with HCC in October 2015. He underwent radiofrequency thermal ablation (RFTA) according to the BCLC classification. Characteristics of Case 2 are summarized in **Table 1**. His immune status was preserved with 623 CD4 cells/mmc, HIV viremia was suppressed (HIV-RNA < 50 copies/mL), whereas transaminases and AFP (20.1 ng/mL) were higher than the normal values. Unresectable recurrence was found in January 2016, and the participant underwent three cycles of trans-catheter arterial chemoembolization (TACE) followed by remission of HCC. At this time point, AFP was found increased (75.6 ng/mL), whereas CD4 cell count was found <500 cells/mmc, and CD4/CD8 ratio was 0.98; CPT was A, and BCLC classification was B. In December 2016, he was treated with direct acting antivirals (ledipasvir plus sofosbuvir) and ribavirin for chronic hepatitis C with sustained virological response. A second recurrence was observed in July 2017, and TACE treatment was re-selected, with a partial response. In 2019, he underwent trans-catheter arterial radioembolization (TARE) with, again, a partial response. His CD4 cell count was <500 cells/mmc, with HIV-RNA undetectable and AFP increased (242 ng/mL). In July 2021, systemic treatment with lenvatinib (12 mg of lenvatinib according to body weight of ≥60 kg) was commenced. Before systemic therapy, his CPT score was A, BCLC classification was B, AST levels were slightly increased (48 IU/L) with normal ALT and bilirubin levels (<59 IU/L and 0.83 mg/dL, respectively), and the immune status was preserved with HIV viremia undetectable. After 1 week of starting lenvatinib, he had decompensated liver disease with CPT score B, and lenvatinib was de-escalated to 8 mg; however, after 2 weeks, the physician decided to permanently discontinue lenvatinib treatment due to progression to end-stage liver disease. ART during the period of observation included an INSTI (i.e., dolutegravir) and nucleos(t)ide analogs (i.e., emtricitabine plus tenofovir alafenamide). In August 2021, the participant was hospitalized for variceal bleeding with endoscopic banding. In November 2021, the participant was still alive with a good control of HIV infection and relatively liver functional reserve (CPT score of B). The participant deceased for the progression to end-stage liver disease in March 2022.

## Discussion and conclusion

PLWH with HCC have a poorer survival outcome than their HIV-negative counterparts, probably because of higher frequency of recurrence and HCC diagnosis at a more advanced stage (6). Consequently, in this setting, HCC is less frequently amenable to curative approaches. The concomitant use of ART and systemic cancer treatment is a complex issue in PLWH, due to known and unknown drug–drug interactions that may result in decreased efficacy of ART and/or HCC progression, as well as potential synergistic toxicity of anti-cancer and ART coadministration. A previous study, although performed in a small number of participants, showed a good safety profile of co-administration of INSTI and sorafenib in PLWH (7). However, in this setting, this evidence is limited to retrospective studies including a small number of participants or case reports of sorafenib for whom efficacy and safety data are like those reported in non-HIV participants (8, 9).

We previously showed that, among participants with advanced BCLC (stage D) and low liver functional reserve (CPT B/C), treatment discontinuation due to adverse events occurred in a minority of participants, suggesting that systemic treatment with sorafenib is well tolerated in PLWH, despite advanced HCC stage (10). Conversely, the presently reported two cases with a good liver functional reserve and an intermediate BCLC score showed low tolerability to systemic treatment with lenvatinib. These two participants were under ART including INSTI and nucleos(t)ide analogs; therefore, potential pharmacokinetic interaction of ART with lenvatinib was unlikely. In conclusions, the presently reported cases underline the need to provide cumulative data on PLWH, addressing the choice between sorafenib and lenvatinib in this setting. A multidisciplinary approach with constant cooperation between HIV and HCC experts is also desirable.

## Data availability statement

The original contributions presented in the study are included in the article/supplementary material. Further inquiries can be directed to the corresponding author.

## Ethics statement

The studies involving humans were approved by Ethics Committee of the San Raffaele Hospital, Milan, Italy. The studies were conducted in accordance with the local legislation and institutional requirements. The participants provided their written informed consent to participate in this study. Written informed consent was obtained from the individual(s) for the publication of any potentially identifiable images or data included in this article.

## Author contributions

GM and CB conceived the idea of publication and wrote the manuscript. CUF and HH participated in the clinical care of the participants and contributed to the manuscript. All authors contributed to the article and approved the submitted version.
